# Shared Mechanisms of GABAergic and Opioidergic Transmission Regulate Corticolimbic Reward Systems and Cognitive Aspects of Motivational Behaviors

**DOI:** 10.3390/brainsci13050815

**Published:** 2023-05-17

**Authors:** Oveis Hosseinzadeh Sahafi, Maryam Sardari, Sakineh Alijanpour, Ameneh Rezayof

**Affiliations:** 1 Department of Animal Biology, School of Biology, College of Science, University of Tehran, Tehran 14155-6465, Iran; oveis1994@m.u-tokyo.ac.jp (O.H.S.);; 2 Department of Neurophysiology, Graduate School of Medicine, The University of Tokyo, Tokyo 113-0033, Japan; 3 Department of Biology, Faculty of Science, Gonbad Kavous University, Gonbad Kavous 4971799151, Iran

**Keywords:** GABAergic interneurons, opioids, signaling pathways, reward system, brain regions

## Abstract

The functional interplay between the corticolimbic GABAergic and opioidergic systems plays a crucial role in regulating the reward system and cognitive aspects of motivational behaviors leading to the development of addictive behaviors and disorders. This review provides a summary of the shared mechanisms of GABAergic and opioidergic transmission, which modulate the activity of dopaminergic neurons located in the ventral tegmental area (VTA), the central hub of the reward mechanisms. This review comprehensively covers the neuroanatomical and neurobiological aspects of corticolimbic inhibitory neurons that express opioid receptors, which act as modulators of corticolimbic GABAergic transmission. The presence of opioid and GABA receptors on the same neurons allows for the modulation of the activity of dopaminergic neurons in the ventral tegmental area, which plays a key role in the reward mechanisms of the brain. This colocalization of receptors and their immunochemical markers can provide a comprehensive understanding for clinicians and researchers, revealing the neuronal circuits that contribute to the reward system. Moreover, this review highlights the importance of GABAergic transmission-induced neuroplasticity under the modulation of opioid receptors. It discusses their interactive role in reinforcement learning, network oscillation, aversive behaviors, and local feedback or feedforward inhibitions in reward mechanisms. Understanding the shared mechanisms of these systems may lead to the development of new therapeutic approaches for addiction, reward-related disorders, and drug-induced cognitive impairment.

## 1. Introduction

Mesocorticolimbic dopaminergic pathways are extensive and encompass various subregions from the VTA to the nucleus accumbens (NAc), the limbic system, and the cortical areas. These neuronal projections are primarily involved in reward, motivational behaviors, and cognitive functions [1]. Due to its reinforcing effects, this system is a potential target for studying different types of drug abuse [2]. It is known that the inhibitory circuits and neuromodulators can affect drug-evoked synaptic plasticity, dependence, and relapse [3]. GABAergic neurons are a significant population of neurons in the ventral tegmental area and project to the NAc or the prefrontal cortex (PFC). They play a crucial role in maintaining the excitation/inhibition balance in reward pathways [4]. The formation of synaptic plasticity at GABAergic-projecting neurons could be vital in processing rewarding or aversive experiences and determining behavioral outcomes [5]. The expression of opioidergic receptors and GABAergic terminals in reward pathways implies their significant role in modulating the neurocircuitry of drug reward [6,7]. Opioid peptides, either endogenous or exogenous, can activate Gi/o-coupled receptors on the GABAergic terminals of the VTA, inhibiting adenylyl cyclase and voltage-gated calcium channels while activating potassium channels and mitogen-activated protein kinase signaling, resulting in the suppression of neurotransmitter release [6]. This eventually disinhibits the dopaminergic neurons of the VTA, activating the reward pathways [8]. The functional interaction of GABAergic and opioidergic systems in different brain regions, such as the hippocampus, amygdala, or prefrontal cortex, indicates their pivotal effects on anxiety, memory, and pain processing [9]. Therefore, it is crucial to understand how these systems work together to mediate reward/reinforcement and how their dysfunction leads to pathological conditions. While many studies have investigated the role of exogenous opiates in reward and addiction for several decades, few have identified the connection between these two systems. In the 1980s, it was found that the administration of opioids increased GABA release in specific brain regions associated with the pain-relieving properties of opioids [10]. More recent research has focused on the complex interactions between the two systems in the context of addiction and withdrawal, although limited research has been conducted on the interactive effects on reward mechanisms at the molecular and cellular levels. This review collects prominent findings in line with our results obtained since 2004 on the interactive role of GABAergic transmission and exogenous opiates in modulating reward, anxiety, and dopaminergic systems in the corticolimbic areas [11,12]. It discusses how their interaction affects the induction of synaptic plasticity in reward pathways, their pathological implications, and potential therapeutic targets for substance use disorders. Furthermore, we attempted to address the unknown molecular mechanisms and hidden links between these two systems by converging discrete results from many studies conducted on opioidergic or GABAergic transmission in cell-specific and region-specific manners. This review discloses the necessity of investigating unexplored areas related to reward and motivational behaviors.

## 2. Neurobiology of Corticolimbic Endogenous Opioidergic Neurons and GABAergic Interneurons

The opioidergic system consists of opioidergic neurons, ligands, and receptors in the central nervous system. Endogenous opioidergic neurons release β-endorphin, met- and leu-enkephalins, dynorphins, and endomorphins as neurotransmitters and neuromodulators [13]. Opioids are widely distributed in the corticolimbic areas, including the PFC, the anterior cingulate cortex (ACC), the hippocampal formation, and the amygdaloid complex, to primarily modulate cognitive functions, the motivator effects of natural and drug rewards, and pain through opioidergic signaling pathways. Opioid receptors, as G protein-coupled receptors (GPCRs), can be divided into the μ-(MOR), κ-(KOR), δ-(DOR), and nociceptin/orphanin FQ (NOP) receptors [14]. Since most of these receptors are coupled with Gi/Go proteins, their stimulation inhibits the activity of adenylyl cyclase, triggering a reduction in the cyclic adenosine monophosphate level to modulate synaptic plasticity, pain processing, and memory- and reward-processing [15,16]. The other signaling pathways, including the activation of β-arrestin2 pathways and the activity of several K^+^ ion channels, have recently been shown to regulate the diverse effects of opioids in the synaptic levels directly or indirectly [17].

β-Endorphin, as a product of the anterior lobe of the pituitary, has stable and long-lasting effects on the amygdala and the hippocampus [18,19]. Hippocampal neurogenesis, emotional behaviors, and stress/anxiety physiology have been speculated to be affected by this peptide, especially via MORs and DORs [20]. Interestingly, β-endorphin enhances the level expression of brain-derived neurotrophic factor mRNA in the hippocampal dentate gyrus (DG) following exercise-induced neurogenesis, which may, through disinhibition of GABAergic interneurons, increase the function of net excitation [21]. Enkephalins, including met- and leu-enkephalins, are highly expressed in the brain reward system to modulate the drug-dependent reward system through opioidergic signaling pathways [22]. Disinhibition of GABAergic neurons through stimulating MORs activates the VTA dopaminergic neurons to trigger motivational behavior. Moreover, the activity of these interneurons was reported to be regulated by dual GABA/enkephalin-mediated inhibitory inputs from the bed nucleus of the stria terminalis (BNST) as an integrative center for limbic information [23]. The other potent endogenous opioids are dynorphins, including dynorphin A, A(1–8), B, α- and β-neoendorphin, leumorphin, and big dynorphin, which bind to KORs to regulate cognitive function and mood disorders under stress [24]. There is a functional interaction between the signaling pathways of dynorphins and GABA in the spinal cord [25] to mediate nociception. The central amygdala (CeA) dynorphin/KOR system also modulates inhibitory GABAergic transmission to mediate drug dependence and reinforcement [26]. Endomorphins also have neurotransmitter and neuromodulator roles in increasing striatal dopamine release [27] and decreasing GABA release in the basal ganglia [28] through binding MORs. The variety of endogenous opioids and their types of receptors, along with the abundant distribution of these receptors in the brain, makes the opioidergic system directly and indirectly involved in corticolimbic physiological and pathological actions. Since the expression of opioid receptors on GABAergic interneuron membranes is very high, the functional interaction of opioids and GABA signaling pathways is of particular importance.

GABAergic inhibitory neurons constitute approximately 10 to 20% of cortical neurons. Cortical GABAergic interneurons have many important functions, including excitatory/inhibitory balance [29], regulation of firing rates, and plasticity [30]. These neurons directly inhibit excitatory cells through feedforward or feedback circuits, or they inhibit stimulation through hyperpolarizing other neurons. Therefore, in neural networks, they can create spatial and temporal modulation of excitability to cause the integration of basic stimuli and higher functions of the cerebral cortex [31,32]. Corticolimbic GABAergic interneurons coordinate cognitive activities, emotional behaviors, and stress/anxiety-related reactions through the release of GABA [33]. Glutamate is the precursor of GABA, which is produced by the action of glutamate decarboxylase enzymes (GAD65 and GAD67); after it is produced, the vesicular transporters (VGAT1 and VGAT2) reuptake GABA for storage in the vesicles for exocytosis [34,35]. GABA transmits its signal via binding to ionotropic GABA_A/C_ and metabotropic GABA_B_ receptors to other neurons or glia. In an adult brain, GABA_A_ receptors are located in neurons and glial cells and induce hyperpolarization by activating these receptors in the target neurons through the influx of chloride ions and the outflow of potassium ions [36]. As a result, the relative threshold of neuronal excitation that fires an action potential is increased, and thus the excitability of neurons decreases [36]. A large number of studies have illustrated a linkage between GABA_A_ receptor activity and addictive disorders such as alcohol [37] as well as nicotine [38] dependence, cannabis abuse [39], and opiate dependence [40], suggesting GABA_A_ receptors are important for brain reward system function. Furthermore, GABA neurotransmission during early development is essential for the morphological maturation of cerebral cortex neurons [41] and synapse formation [42]. Corticolimbic GABAergic dysfunction, including any disturbance of GABA synthesis, transport, GABA receptor expression, and/or GABA inactivation, was reported to happen in various nervous disorders [43,44].

Opioids regulate GABAergic function via binding to presynaptic MORs and DORs of interneuronal terminals to decrease GABA release in the hippocampus, the amygdala, and the cortical areas [6,7,45,46]. Moreover, postsynaptic opioidergic signaling pathways were suggested to hyperpolarize GABAergic interneurons to reduce the spontaneous GABA-mediated synaptic input to other neurons, including dopamine neurons [47].

## 3. Neuroanatomical Studies of Corticolimbic GABAergic Interneurons and Opioidergic System in Reward-Related Mechanisms

The corticolimbic system, which consists of the prefrontal cortices, the hippocampus, and the amygdala, processes higher cognitive functions, including memory formation, decision-making, and emotional regulation [48,49]. Dysfunction of GABAergic interneurons, which mediate regulation and coordination of cortical pyramidal neuron activity [50], may be involved in epilepsy [51], schizophrenia, and anxiety [52]. Enkephalins, endorphins, and dynorphins and opiate drugs such as morphine and heroin act through MORs, KORs, and DORs which are widely distributed at the pre- and postsynaptic sites in the cortex and limbic area [53]. Opiates produce their disinhibitory effect in the hippocampus and the NAc mainly via modulation of GABA-containing neurons [54,55]. Coexpression of MOR1 and GAD67 on the NAc neurons (Table 1) indicated a direct interaction between opioidergic and GABAergic systems in reward processing [56].

### 3.1. Prefrontal Cortex

The human prefrontal cortex consists of many subdivisions, including the dorsomedial prefrontal cortex (dmPFC), the ventromedial prefrontal cortex (vmPFC), the ventrolateral prefrontal cortex (vlPFC), and the orbital frontal cortex (OFC). The ACC is also considered a part of the PFC [57]. Preclinical and clinical studies have indicated that the prefrontal GABAergic interneurons have key roles in the control of social interaction behaviors [58]. In a study conducted by Liu et al. (2020) in mice, the role of two major prefrontal GABAergic interneurons, parvalbumin (PV)- and somatostatin (SST)-expressing interneurons, was assessed in social behaviors. The authors reported that the synchronized activation of PV- or SST-interneurons at low gamma frequency improved social interaction behaviors. Furthermore, suppressing these interneurons induced a reduction in low gamma power and impaired mice sociability [59]. The medial PFC (mPFC) is one of the major inputs to the reward system, and it was reported that a lesion in infralimbic subregion of the mPFC prevented morphine-induced conditioned place preference (CPP) [60]. Additionally, the opioidergic system induces changes in the expression of glial fibrillary acidic protein in the PFC through the activation of GABAergic transmission from the mediodorsal thalamic nucleus [11]. Taki and colleagues used an immunolabeling method to indicate that MORs were expressed in the PFC GABAergic neurons. The PFC interneurons also release preproenkephaline to bind to MORs for triggering signaling pathways [61]. MOR signaling was reported to suppress voltage-dependent Na^+^ currents by the recruitment of the protein kinase A and protein kinase C (PKC) pathway in the PFC non-pyramidal neurons [62]. Using whole-cell patch clamp, the ventrolateral orbital cortical (VLO) MOR activation decreased the frequency, but not the amplitude and miniature inhibitory postsynaptic currents (mIPSCs), suggesting that the presynaptic GABA suppression mediates the VLO MOR effects [7]. Neuroimaging studies indicated that amphetamine and alcohol administration resulted in endogenous opioid release in the frontal lobe and the OFC in humans [63,64]. Interestingly, intermittent access to food produced a neuroadaptation in the mPFC opioid system to induce binge-type eating as an addiction-like disorder [65].

### 3.2. Hippocampus

The hippocampus is a part of the limbic system which is involved in learning and memory functions, specifically episodic and contextual memory, reward and social memory [66], reward processing [67], and drug reinforcement [68]. Classically, the hippocampus refers to the DG and the cornu ammonis (CA) subfields, including the CA1, CA2, and CA3 regions. It should be considered that opioid receptors are expressed in the entire hippocampus [69]. GABAergic interneurons make up approximately 11% of cells in the CA1 region of 30-day-old Wistar rats [70]. Considering GABAergic transmission has a determinant role in the regulation of hippocampal pyramidal neuronal input and output, it seems that the activity of GABAergic interneurons is essential in controlling hippocampal memory performance [71]. PV- and SST-expressing interneurons are two interneuron classes that mainly exist in the CA1 region. These interneurons also control the information flow from internal (CA3) and external (entorhinal cortex) regions of the hippocampal formation. PV-expressing interneurons regulate the timing of principal neuronal spiking, while SST-expressing interneurons control the principal neuronal spiking magnitude [60]. MORs are expressed postsynaptically on the soma and the dendrites or presynaptically on the axonal component of GABAergic interneurons in the CA1 region and the DG (Table 1) [72]. Hippocampal MORs are mainly expressed on the synaptic terminals of interneurons which inhibit pyramidal cells, and also, the receptors may be expressed on a limited number of interneurons to form synapses with other interneurons [73]. In the hippocampus, the activation of MORs and DORs localized on GABAergic interneurons suppresses GABA release to promote excitatory neuronal transmission via disinhibiting principal neurons [9]. In addition, MORs are highly expressed in the soma of astrocytes in the CA1 region (Table 1) [74]. Pharmacological and electrophysiological studies confirmed that MOR activation is necessary for the formation of long-term potentiation (LTP) in the DG but not in the CA1 region [75], while KOR activation prevented the induction of LTP in the DG [76]. Utilizing MOR knockout mice, it was found that the MOR signaling pathway in GABAergic interneurons of the ventral hippocampus (VH) is necessary for the antidepressant effect of tianeptine [77]. Interestingly, nicotine administration induced a synaptic potentiation in the DG that was associated with a diminished local GABAergic circuit for disinhibiting granule cells to induce nicotine-induced synaptic potentiation and drug-associated memories [78]. Evidence suggests the important role of GABAergic interneurons in the regulation of information processing. For example, it was illustrated that PV- and SST-expressing interneurons of the CA3 region and the DG differentially modulate the information flow through the hippocampal circuit [79].

### 3.3. The Amygdala

The amygdaloid complex is involved in emotional and motivational behaviors as well as in processing fearful and rewarding environmental stimuli [80]. This complex consists of numerous nuclei, including the cortical, the basolateral (BLA), and the central (CeA) nuclei, which are associated with the cerebral cortex and the striatum [81,82]. Amygdala MORs modulate anxiety [83], stress responses, and social behaviors [84]. Using MOR knockout mice, it was found that the inactivation of the BLA MORs diminished the cued recall of reward memories [85]. The microinjection of MORs or GABA_A_ receptor antagonists into the CeA reduced ethanol-maintained response in rats [86]. CeA KOR activation not only decreased GABAergic inhibitory postsynaptic currents (IPSCs) but also controlled GABA transmission at presynaptic sites, indicating the implication of KOR signaling in the tonic inhibition of GABAergic neurotransmission in the CeA [87]. An acute low dose of ethanol increased the CeA GABA_A_ receptor-mediated inhibitory postsynaptic potentials (IPSPs) and currents (IPSCs) and also augmented GABAergic transmission at the pre- and postsynaptic levels [88]. MORs are expressed in high density in the BLA, especially in the somatodendritic sites of pyramidal neurons and interneurons [89]. Given that the majority of the BLA interneurons release GABA on pyramidal neurons, the activation of interneuronal MORs reduces GABA release to disinhibit the pyramidal projection neurons [90]. Moreover, microinjection of a GABA_A_ receptor agonist into the BLA decreased morphine-induced reward [12], showing that the BLA GABAergic and opioidergic systems may interact in reward processing.

**Table 1:** Colocalization of opioid receptors and GABAergic markers in the corticolimbic regions.

Opioid Receptor	GABAergic Marker	Brain Region
κ-opioid receptors	Glutamate decarboxylase [91]	Hippocampus [92]
κ-opioid receptors	Calretinin [93]	Dorsal striatum [93]
κ-opioid receptors	Parvalbumin [94]	Basolateral amygdala [94]
δ-opioid receptors	Somatostatin [95]	Dentate gyrus [95]
δ-opioid receptors	Parvalbumin [96]	Hippocampal CA1 [96]
δ-opioid receptors	Parvalbumin [97]	Hippocampal CA2 [97]
δ-opioid receptors	Somatostatin [98]	Prelimbic cortex [98]
δ-opioid receptors	Parvalbumin [98]	Prelimbic cortex [98]
δ-opioid receptors	Calretinin [93]	Nucleus accumbens core [93]
μ-opioid receptors	Glutamate decarboxylase [23]	Ventral tegmental area [23]
μ-opioid receptors	Glutamate decarboxylase [73]	Dentate gyrus [73]
μ-opioid receptors	Parvalbumin [73]	Dentate gyrus [73]
μ-opioid receptors	Somatostatin [73]	Hippocampus (OLM) [23]
μ-opioid receptors	Somatostatin [99]	Dentate gyrus [99]
μ-opioid receptors	Parvalbumin [100]	Hippocampal CA1 [100]
μ-opioid receptors	Parvalbumin [101]	Orbitofrontal cortex [101]
μ-opioid receptors	Calbindin-D28K [102]	Dorsal Striatum (DA neurons) [102]
μ-opioid receptors	Calbindin-D28K [96]	Nucleus accumbens core (MSNs) [96]
μ-opioid receptors	Vasoactive intestinal peptide [103]	Neocortex [103]
μ-opioid receptors	Vasoactive intestinal peptide [104]	Anterior cingulate cortex (L1) [104]
μ-opioid receptors	Somatostatin [104]	Anterior cingulate cortex (L5 and 6b) [104]
μ-opioid receptors	Calretinin [105]	Central amygdala [105]
μ-opioid receptors	Glutamate decarboxylase [56]	Nucleus accumbens [56]
μ-opioid receptors	Parvalbumin [106]	Hippocampal CA3 [106]

Abbreviations: MSN, medium spiny neuron; CA, cornu ammonis; OLM, oriens lacunosum moleculare; L, layer.

## 4. Opioid and GABA Receptor Dynamics in Motivational Behaviors

In rodents, the hippocampus has a high density of DORs and low expression of MORs. High levels of MORs and DORs and low levels of KORs were reported in the rat amygdala. The PFC contains a low density of MORs and KORs, while DORs were found to have a high density [107]. Brain GABA_A_ receptors are mainly composed of five subunits, including two α-subunits, two β-subunits, and one γ- or δ-subunit, which originate from different genes (α1–α6, β1–β3, γ1–γ3, δ, ε, θ, π, or ρ1–3). The DG granule cells express GABA_A_ receptors containing α4-, α5-, α6-, and δ-subunits at extra- and perisynaptic locations in mice [108].

A single administration of morphine diminished the GABA receptor binding site in the cortex. However, chronic administration increased the binding affinity, suggesting that the GABAergic system modulates morphine effects [109]. Acute administration of morphine results in a decrease in the NAc NMDA-induced GABA release via the MOR signaling pathway [110]. Sasaki et al. (2002) reported that the activation of MORs by morphine had an anxiolytic-like effect in female mice. This response was associated with [(3)H] muscimol binding augmentation in the mediodorsal thalamic nucleus and the amygdala, but not the hippocampus [111]. DAMGO, a selective MOR agonist, reduced evoked excitatory postsynaptic currents (eEPSCs) and also excitability in GABAergic interneurons; the last effect was mediated through the outward current of K^+^ channels to control pain transmission in the spinal dorsal horn [112]. Enhancement of mesolimbic GABA transmission through GABA_B_ receptors, blocked heroin reinforcement, suggesting that GABA agents can be used in the treatment of opiate abuse [113]. MOR and DOR signaling pathways inhibit the CA1 PV-expressing interneurons by occlusion of potassium channels at somatodendritic postsynaptic sites and calcium channels in the presynaptic terminal (Table 1) [114].

Opioids and GABA are evenly involved in anxiety and reward-related behaviors [115,116]. Enhancement of opioid signaling due to ethanol intake has been shown to increase GABAergic transmission by potentiating the release of GABA from presynaptic terminals and increasing the activity of GABA_A_ receptors in the VTA [117,118]. This leads to an increase in the inhibitory tone of VTA networks following a reduction in pain and anxiety. In contrast, opioids inhibit GABA release from presynaptic terminals of interneurons located in the rat hippocampus [46], which suggests that opioids and GABA interaction can be highly dissociated contingent upon the brain region and disorder. GABA modulates the activity of opioid receptors by increasing their sensitivity to acute action of opioids during withdrawal. Interneurons expressing MORs in the NAc are known to play a critical role in regulating opioid reward. Activation of these neurons inhibits dopamine release, which can attenuate the rewarding effect of opioids [119]. The interaction of opioidergic and GABAergic systems in the NAc is bidirectional, contributing to the modulation of opioid reward through GABAergic interneurons of the VTA and the NAc [120]. There is a significant expression of KOR-containing synapses on the rostrocaudal axis of the VTA and the substantia nigra [121,122]. KORs are localized in somatodendritic terminals of dopaminergic neurons, particularly D1R-containing medium spiny neurons (MSNs), as one of the dopaminergic midbrain inputs for dynorphin in compulsive behavior. Noteworthily, the local inhibition through D1R-MSNs is mediated by GABA rather than KORs, whereas the blockade of KORs does not change the net inhibitory impact [123]. However, enhancing the activity of KORs in the VTA reduces the spontaneous firing rate in dopaminergic neurons [124]. In the NAc MSNs, MOR knockout leads to a significant reduction by exerting its effect on presynaptic potassium channels, proto-oncogene tyrosine-protein kinase Src, and β-arrestin2 [8,125]. In the ventral pallidum, MORs block GABAergic inputs to both dopaminergic and non-dopaminergic neurons following changes in reward behavior [126].

In addition, the hyperpolarization of MORs can invoke both local and NAc-projecting neurons of the VTA [127], while local GABAergic neurons are abruptly desensitized following the activity of MORs. Importantly, potentiating the activity of MORs hyperpolarizes a subpopulation of non-pyramidal neurons in contrast with DORs. Adversely, GABAergic neurons of the anterior agranular part of the insular cortex express KORs, which play an important role in disinhibiting pyramidal cell inputs of layer 5 onto the substantia nigra [128]. Furthermore, it should be mentioned that dynorphin reduces GABA release via regulating MORs in pyramidal cells of layer 5 while increasing glutamate release, implicating the disinhibition of the local circuit [129]. Remarkably, the activation of presynaptic MORs has a huge impact on reducing inputs of the fast-spiking interneurons (FSIs) to other FSIs or non-FSI neurons, counter to the pyramidal cells. Despite this, triggering DORs leads to a reduction in FSI inputs onto pyramidal neurons and other adjacent fast-spiking interneurons (FSIs); however, it has no significant effect on GABAergic transmission from non-FSI neurons. Additionally, the activity of KORs does not affect the inputs of FSI to other neurons of the insular cortex. It is worth mentioning that in the mPFC, DOR-expressing neurons opposing MORs enhance GABAergic transmission from SST-expressing interneurons to PV-expressing interneurons (Table 1), resulting in the disinhibition of pyramidal neurons [98]. In the ventrolateral part of the orbitofrontal cortex (OFC), presynaptic MORs suppress GABA release onto pyramidal neurons in rats. Comparable to the role of MORs in regulating FSIs of the mPFC, MOR-induced LTD in the presynaptic PV-expressing type of FSIs blocks GABAergic input onto pyramidal neurons of the medial OFC [7,130]. In hippocampal CA1, as one of the prominent gateways of the corticolimbic reward system, opioidergic modulation hyperpolarizes the local GABAergic interneurons of the CA1 subregion, which decreases GABAergic inputs of interneurons and neuroglia-form cells mediating feedforward inhibition, to the pyramidal cells [106,131,132,133,134]. In the CA1 region, DORs can also suppress GABA transmission, even though they are not the primary regulators of this mechanism [131]. Feedforward and feedback inhibition are known to be important mechanisms in local microcircuits of the hippocampus, particularly the CA1 subregion, and MORs diminish both of these forms of inhibition, unlike DORs. MORs and DORs similarly block the spontaneous GABA release, conversely to the monosynaptic type of inhibitory postsynaptic potentials (IPSPs) [131]. One of the captivating findings in the neuroanatomical distribution of opioid receptors in the CA1 region showed that MORs suppress GABAergic interneuron inputs onto the soma, while DORs suppress the inputs to the dendrites of pyramidal neurons [9]. Corroborating this, a recent study manifested that MORs block FSIs but not the inputs of non-FSI basket cells to CA1 pyramidal neurons [100]. CA2, as the crucial subregion of the hippocampus for social behavior and novel memories, is not exempt from opioidergic modulation. DORs presynaptically induce LTD at the PV-expressing type of FSI innervating solely to the CA1 pyramidal neurons and short-term depression in the CA1 pyramidal neurons [97]. In the CA3 region, MORs mediate the disinhibition of GABA transmission (Table 1), which increases the net inhibitory activity, although DORs and KORs do not contribute to this mechanism [106,135]. The DG is also implicated in the same disinhibitory mechanism as that in the CA1 and the CA3 regions. The DG opioid receptors, particularly MORs and DORs, generate disinhibition due to their impact on GABAergic neurons, even though this disinhibition is less effective on LTP induction via MOR suppression of GABA_A_ and GABA_B_-mediated IPSCs at the synapses of DG granule cells compared to the CA1 and the CA3 regions [100]. It is important to note that improving the activity of KORs constrains glutamatergic transmission from the inputs of perforant pathways independent of GABA transmission [6]. KOR activation inhibits glutamate transmission from perforant path inputs without affecting GABA transmission. KORs interfere with the activity of the DG hilar mossy fibers by inhibiting LTP, which is possibly mediated by GABA_A_-dependent mechanisms [136]. Interestingly, KORs inhibit the BNST presynaptic GABA release [137]. Furthermore, the neurons projecting from the NAc to the VP densely express GABA and enkephalin, along with the coexpression of MORs. The activity of MORs in the VP contributes to reward-related mechanisms such as hedonic responses to alcohol intake, seeking behavior reinstatement, and palatable food. MORs modulate GABA neurotransmission in the VP during cocaine seeking, whereas stimulating these receptors decreases extracellular GABA and suppressing their activity disinhibits GABA release in the VP. Conclusively, the withdrawal following cocaine seeking causes an elevation in enkephalin levels of the VP, which results in the impairment of MOR activity in GABAergic neurons, suggesting the contribution of withdrawal difficulties in addicts who try to resist relapse [138].

## 5. Neurochemical Markers for the Colocalization of Opioidergic Neurons and GABAergic Interneurons

The different isoforms of GAD, including GAD65 and GAD67, are commonly used as neurological markers to detect GABAergic neurons in terms of their activity [139]. Understanding the structural and functional properties of GABAergic and opioidergic neuronal markers in parallel leads researchers to decipher new inhibitory/modulatory circuits responsible for opiate-induced reward mechanisms in various brain regions. The endogenous opioid neuropeptides, including enkephalins and dynorphins, activate MORs and DORs to inhibit neuronal excitability and synaptic output [6]. The DG MORs and DORs predominantly exist in a distinct subpopulation of GABAergic interneurons to exert a suppressive effect on the granule cells, resulting in the regulation of LTP. Traces of dynorphins and KORs are abundantly located on the mossy fibers of the granule cells and the dendrites at lower levels. Likewise, KORs have been localized to the perforant pathway terminals and supramammillary afferents to granule cells. Evidence suggests that opioids regulate the excitability of other hippocampal mechanisms, including adult gonadal hormones, neurogenesis, and rewarding responses, through the interactive effect of GABAergic interneurons and the opioidergic system [95]. Hippocampal GABAergic interneurons send their inputs to opioidergic neurons stabilizing neurophysiological mechanisms of motivation and antidepressant effects through MORs [77]. Therefore, the colocalization of specific GABAergic and opioidergic markers sheds light on the underlying GABAergic mechanisms of reward-related behaviors and local neural microcircuit tracing.

### 5.1. Opioids and Glutamate Decarboxylase 65/67

Glutamate decarboxylase (GAD67), as an enzyme that catalyzes the conversion of glutamate to GABA, is expressed by a subset of inhibitory neurons, including PV- and SST-expressing interneurons [140]. Interestingly, prefrontal cortical interneurons expressing GAD65/67 are more active than the ones lacking GAD65/67 [141]. Neuronal circuit tracing and immunostaining studies have widely utilized antibodies and anti-GAD67 plasmids to locate the active GABAergic neurons in the mesocorticolimbic regions, including the VTA involved in reward-related behaviors [142]. GAD67 may interact and colocalize with the opioid system [56]. Opiates affect lateral paracapsular interneurons of the amygdala, exerting feedforward inhibition onto the BLA GABAergic circuits by reducing GABA release [143]. Opioid receptors moderate the activity of GAD67 in the hippocampus [92], following changes in the levels of GABA released from presynaptic terminals (Table 1). This modulation may be involved in antidepressant-related mechanisms through KORs [91], addiction (dependence on opioid drugs), and anxiolytic mechanisms, particularly in the prelimbic cortex and the amygdala [144]. Calaj et al. (2020) indicated that GAD67-expressing interneurons in the VTA, a key area involved in reward processing, may also modulate the activity of the opioid system through their GABAergic inhibitory inputs [145]. This study also suggested that the MOR gene, *Oprm1*, is vastly expressed in 50% of GABAergic neurons in the substantia nigra pars reticulate (SNr), 30% in the VTA, and 70% in the rostromedial tegmental nucleus of male rats. MOR activation has a critical role in regulating reward behaviors to disinhibit the VTA dopaminergic neurons via suppressing GABAergic neurons expressing GAD67 (Table 1) [23]. However, the neurons projecting from the VTA to the hippocampal DG release GABA and glutamate [146]. It should be considered that the VTA neurons coordinate the reactivation of hippocampal spatial memory formation in reward-related learning [147]. It seems that opioidergic modulation of the VTA GABAergic neurons expressing GAD67 may change the outcome of reward mechanisms exclusively in the CA1 region and DG corticolimbic areas, although there are not many studies for confirmation. MORs expressed broadly in PV basket cells are widely distributed in the hippocampus, where they are localized at the presynaptic terminals of the GABAergic interneurons to transmit a disinhibitory effect on pyramidal cells following the modulation of analgesia, reward, and euphoria. Furthermore, MOR activation of hippocampal astrocytes induces glutamate release, which is regulated by local interneurons, to enhance the excitability of presynaptic axon terminals in Schaffer collateral-CA1 synapses following the induction of synaptic plasticity [148]. Hippocampal MORs are commonly expressed on interneurons specific for suppressing pyramidal cells and exist on a restricted number of interneurons to target other interneurons. It is noteworthy that the DG MORs are extensively colocalized with GAD^+^ parvalbumin, but not somatostatin (SST) interneurons [73,99].

### 5.2. Opioids and Somatostatin

The brain SST-expressing interneurons interact with the opioid system to regulate the perception of pain, reward, and other physiological processes [149,150,151]. These interneurons are found in many brain regions, including the hippocampus, the neocortex, and the striatum [152,153]. SST-expressing interneurons undergo LTP to shape hippocampal output during different physiological activity patterns. SST-induced synaptic plasticity relies on the activation of T-type calcium channels to designate synapse specificity. Since SST interneurons preferentially target the distal dendritic regions of the CA1 pyramidal neurons, SST-induced LTP prioritizes the excitatory inputs coming from the entorhinal cortex and the CA3 region [140]. MOR-like immunoreactivity is present in SST-expressing oriens lacunosum moleculare (OLM) interneurons innervating to the distal dendrites of hippocampal pyramidal cells, although the DG MORs are not colocalized with SST-expressing interneurons extensively [73,99]. These findings suggest that SST-induced plasticity likely undergoes the impact of downstream signaling of MORs in the presence of endogenous or exogenous opioids and reward. SST-expressing interneurons are densely expressed in the limbic areas to play a crucial role in modulating emotional and stress responses [154]. These interneurons in the VTA modulate the activity of dopaminergic neurons through their inhibitory inputs to modulate the corticolimbic rewarding mechanisms. The inhibitory inputs may regulate the release of dopamine in response to opioid receptor activation to mediate reward-related behaviors [155]. The VTA SST-expressing interneurons are involved in the regulation of reward-related behaviors via increasing dopamine release during the activation of opioid receptors [6,156]. Considering the most common interneuron subtypes in the VTA are SST subtypes, it can be suggested that these interneurons have an important impact on the modulation of the midbrain reward pathway [157]. Moreover, hippocampal SST-expressing interneurons modulate the release of opioid peptides [158], thus influencing the perception of pain and possibly the reward system. The opioidergic system changes hippocampal excitability, LTP, and epileptic activity through local and long-projecting interneurons. Two major DG endogenous opioids are enkephalins and dynorphins, which take part in circuit modulation by imposing contradictory impacts on neuronal excitability. In the DG, enkephalins predominantly bind to MORs and DORs, while dynorphins are specifically coupled with KORs. Enkephalins affect the mossy fiber pathways into the lateral perforant pathway (PP) and in a subpopulation of SST-expressing interneurons. Hippocampal interneurons decrease the release of enkephalins when opioid receptors are activated to regulate pain perception [95]. Moreover, they can arguably modulate reward-related mechanisms; however, this has yet to be found. Accordingly, it is expected to find SST+ interneurons and opiate receptors in the corticolimbic regions involved in the reward mechanisms.

### 5.3. Opioids and Parvalbumin

Parvalbumin (PV) is a protein expressed in inhibitory interneurons; with high calcium-binding affinity and low molecular weight, it is abundant in the corticolimbic areas [159]. Opioids and parvalbumin interneurons are extensively colocalized in the corticolimbic regions. Using opioid neuropeptides which are photoactivable, it has been shown that DORs modulate the response to the enkephalin ligands and its kinetics, while they consecutively affect hippocampal CA1 PV-expressing interneurons through the low expression of MORs; however, MORs and DORs interact independently of the signaling pathway of PV-expressing basket cells (BCs), and this modulation does not change the synaptic transmission or the somatodendritic potassium currents [114]. MORs are broadly localized at GABAergic presynaptic terminals of interneurons to transmit a disinhibitory impact on hippocampal pyramidal cells as well as the presence of MORs on the astrocytes to increase the excitability of presynaptic axon fibers of the Schaffer collateral CA1 synapses via glutamate [148]. Astrocytic MOR signaling may be involved in adult DG neurogenesis, seizure, stress-induced memory impairment, and opioid-associated reward processing [148]. In addition, enkephalin induces outward somatodendritic currents in PV-BCs predominantly by DORs rather than MORs. These findings indicated that despite the inhibitory effect of MORs and DORs on the CA1 PV-expressing basket cells via suppressing GABA release by the inhibition of voltage-sensitive Ca^2+^ channels, the colocalization of MORs and DORs on the PV-expressing interneurons does not necessarily imply the functional crosstalk between these two potassium channels [114]. It is worth mentioning that GABA released from the terminals of the PV-expressing interneuron onto the CA1 pyramidal neurons is extensively suppressed by MORs (Table 1) [160]. In the hippocampal CA2 region, PV-expressing interneurons express a distinct long-term depression (LTD) through feedforward inhibition of the CA3 Schaffer collateral inputs to the CA2 region which is regulated by the activity of DORs. Enhancement in the activity of MORs via a DAMGO agonist increased excitatory postsynaptic potential (EPSP) amplitude and decreased the time in the deeper area of the CA1 in contrast with PV-expressing interneurons mediating feedforward inhibition via DORs from the CA2 pyramidal cells which also increased action potential firing in deep pyramidal cells [97,161]. It is important to note that hippocampal CA1 and CA2 regions are significantly involved in reward-related behaviors and social memory, respectively [162]. Furthermore, hippocampal–thalamic afferents diversely target specific synapses to the PV-expressing interneurons of the infralimbic frontal cortex in mice [163], and thalamostriatal projections to the NAc PV-expressing interneurons form AMPAR-enriched synapses which are crucial for inappropriate reward-seeking behaviors and mediated by the opioidergic system. This mechanism is sufficient for dampening sucrose seeking regardless of behavioral phenotypes. Morphine exposure potentiated SST inhibitory inputs onto PV-expressing interneurons of the prelimbic cortex following disinhibition in pyramidal cells through upregulating DOR-dependent Rac1 receptors in SST-expressing interneurons to enhance reward [98]. Noteworthily, hippocampal CA1 interneurons expressing MORs enhance the activity of fibers in the Schaffer collaterals resulting in phase coupling between CA3 γ and CA1 γ oscillations. Gamma oscillation of CA3 triggers CA1 gamma following suppression of the intrinsic fast gamma of CA1. This synaptic modulation can switch the low γ frequency into high γ frequency in the CA1 network, which controls the flow of information between the medial entorhinal cortex, the CA3, and the CA1 regions. Interestingly, the activation of MORs decreases gamma frequency in both the CA1 and CA3 regions. The gamma coupling in these regions shows the importance of MORs in modulating phase coupling of the information flow in reward-related mechanisms [164]. In addition, neuronal projections from the mOFC to the BLA are partially controlled by the opioidergic system, contributing to reward mechanisms. MORs suppress synaptic transmission of GABAergic neurons, specifically PV interneurons, onto the OFC pyramidal cells with amygdala selectivity (Table 1) [101]. On the other hand, an intermittent chronic ethanol administration-induced reward changes the function of the BLA rewarding system via KORs in male rats (Table 1) but not in females [94]. Taken together, these findings reveal that the corticolimbic areas are extensively involved and integrated into the opioidergic system and PV-expressing interneurons regulating reward. Additionally, thalamostriatal and thalamic–hippocampal innervations to the PV interneurons are required for suppressing reward-seeking behavior which is rapidly disengaged by endogenous or exogenous opioids [165]. Moreover, endogenous opioidergic transmission modulates the PV morphology and SST interneuronal dendrites in the mPFC. Interestingly, chronic morphine administration increases the length of dendrites and their complexity. It is important to note that these changes are long-lasting until seven weeks after blocking morphine [166]. In a study utilizing transgenic mice for knocking out MORs in MOR-flox mice crossed with the Cre line and under the expression of a VGAT promoter to specifically ablate MORs on the hippocampal interneurons, tianeptine-induced rewarding features indicated the preference for the tianeptine-paired side in a conditioned place preference (CPP) task, showing that the ablation of MORs in interneurons has a huge impact on reward-related behaviors. According to the fact that changes in reward processing are the principal characteristics of depression, it is known that the expression of MORs on dopaminergic type-1 medium spiny neurons regulates the rewarding impact of morphine, as assessed by CPP. Tianeptine rectifies the dysregulation of corticolimbic regions, including the PFC, the ACC, the hippocampus, and the amygdala, through alteration in interneuronal MOR signaling cascades, rather than inducing euphoria and restoring reward [77].

### 5.4. Opioids and Calbindin-D28K

Calbindin-D28K is a protein that buffers the intracellular levels of calcium in inhibitory interneurons [167]. This protein is expressed in a variety of interneurons in the mesolimbic reward pathways. Nevertheless, few studies show the colocalization of corticolimbic opioid receptors and calbindin. Since the focus was mainly on the striatum in the past years, we cannot deny the importance of the striatum and its connections to the corticolimbic regions driven by reward information. The striatum, as the central hub of this pathway, modulates motivation and sensorimotor information [102]. The matrix of striatal tissue is enriched at 85–90%. In contrast, some areas of the striatal tissue with extensive presence of MORs have lower expression of calbindin [168]. In this pathway, afferent neurons innervating to the neurons in the medial shell of the NAc differently express calbindin as a boundary marker to specify subregions and separate the core and shell of the NAc [169]. Aldehyde dehydrogenase 1A1, an enzyme contributing to the synthesis of retinoic acid (RA), plays a significant role in regulating the expression of MORs, and aldehyde dehydrogenase 1A1-positive axons predominantly send their fibers to the dorsal striatum. The absence of this enzyme results in a substantial decline in the MOR expression level of the dorsal striatum; however, it does not increase calbindin levels. This enzyme moderates the dorsal striatal postsynaptic MOR expression through retinoic acid signaling [170]. Dopaminergic neurons of this pathway contain calbindin-D28K to regulate the mesocorticolimbic inputs to the mPFC (Table 1) [102]. Similarly, sucrose consumption, as a reward, resulted in lateralized depletion in MORs and dopamine D2 receptors on the NAc medium spiny neurons [96], whilst the dorsal striatum and the NAc core exhibited dense calbindin expression in contrast with the shell [96]. In these studies, anti-calbindin antibodies were used to distinguish the subregions of the striatal matrix in particular postsynaptic striatal neurons. In drug addiction mechanisms, the paraventricular thalamic nucleus plays a critical part in modulating goal-oriented behaviors as a key hub of neural circuits in addiction. The paraventricular thalamic neurons are primarily glutamatergic neurons that are immunoreactive to vesicular glutamate transporter 2; despite that, paraventricular thalamic neurons lack local GABAergic interneurons like the rest of thalamic nuclei. Conversely, calbindin and calretinin are strongly expressed in this nucleus, implying the presence of cortical long-projecting GABAergic inputs [171]. Recently, it has been suggested that the lateral habenula (LHb) affects negatively motivated behaviors following major depression. This region is known to be an antireward center due to its various inputs from the limbic forebrain and the basal ganglia. Calretinin and calbindin are the only calcium-binding proteins and interneuron markers selectively expressed in the LHb [172].

### 5.5. Opioids and Vasoactive Intestinal Peptide

Vasoactive intestinal peptide (VIP) is a neuropeptide that has been found to modulate opioid signaling in numerous brain compartments, including hypothalamic and extrahypothalamic regions and the suprachiasmatic nucleus [173,174]. Although there are very limited studies investigating the role of VIP interneurons and the opioidergic system in reward, an immunohistochemical study manifested that MORs are highly expressed in the VIP interneurons of the neocortex [61]. Interestingly, VIP is highly expressed amongst the hyperpolarized interneurons with adapting spiking patterns in response to DAMGO [175]. A fraction of hippocampal CA1 VIP interneurons distinctly integrates into the goal-oriented behaviors by adapting their activity to correspond with the activity pattern of pyramidal neurons. Whereas the CA1 pyramidal neurons may be modulated by endogenous opioids, VIP neurons of this region exert their modulatory impact near the location of the learned objective, which is necessary for reward-related shifting of pyramidal cells reorganizing the CA1 reward microcircuit [176]. There is a distinct subpopulation of VIP-expressing cells within the VTA dopaminergic neurons which are also enriched in calbindin [177]. Optogenetic activation of VIP interneurons in the prelimbic (PL) and infralimbic (IL) regions of the mPFC reduced high-calorie food intake, while it is partly mediated by opioid receptors and the reward system [178]. VIP-expressing subiculum-innervating cells express genes involved in the signaling of opioid neuromodulators such as Oprd1 and Oprl1 in the hippocampal CA1 region [179]. The level of inhibitory interneurons expressing MORs in the ACC depends on the layer, ranging from 1.3% to 13%. The highest expression of VIP interneurons in the ACC is in L1, and the lowest is in L6a. In addition, SST-expressing interneurons are moderately expressed in L6b which identically colocalizes with MORs. Accordingly, the preprodynophin mRNA is vastly expressed in BIP interneurons and pyramidal cells of L6b; thus, SST-expressing interneurons colocalizing with MORs in L2/3 and L5 release dynorphins as well as MOR-expressing pyramidal cells of L6b. Moreover, following the presence of dynorphins and enkephalins, MORs extensively settle in L5 and L6b of the ACC (Table 1) [104]. Remarkably, morphine intake voluntarily strengthens mRNA levels of opioid receptors in the PFC but does not change VIP levels; however, the result in the NAc was contradictory with a slight elevation in mRNA level of VIP in morphine-administered rats [180].

### 5.6. Opioids and Calretinin

Calretinin or calbindin 2 is a protein akin to the calbindin D28K which is essential for calcium signaling. This protein is abundantly expressed in the amygdala and the hippocampus. In the lateral nucleus of the human amygdala, neuronal terminals expressing calretinin form synapses with calbindin-expressing neurons. In addition, calretinin and calbindin structural colocalization are evident in prefrontal–hippocampal circuitry; therefore, they modulate the reward system [181]. Colocalization of calretinin and opioids suggests that calretinin modulates opioid receptors’ response to endogenous and exogenous opiates. Furthermore, calretinin can regulate the release of dopamine in response to the reward-induced opioidergic system [182]. The exact mechanism by which this occurs is not well understood, but it is thought to involve changes in calcium signaling of GABAergic interneurons [183]. Notably, in the CeA, a large proportion of interneurons express enkephalins and MORs, while they also express calretinin in salt overconsumption (Table 1) [105]. A large number of calretinin- and substance P-expressing neurons are located in the shell of the NAc, while enkephalins, calbindin, and GABA receptors are mainly present in the core. In the NAc, GABA_A_ receptors, via their interaction with GABA_B_ activity, may result in the disinhibition of local GABA signaling. Blockade of dopaminergic neurons in this region might be due to the enhancement in the activity of GABA_B_ receptors following dampened activity of DOR 1 and 2 in the NAc GABAergic neurons, especially the calretinin-expressing interneurons. In addition, inhibition of the VTA KORs blocks the D1-MSN response to prolonged stimulation (Table 1). On the contrary, inhibiting DORs hinders behavioral phenotypes induced by the D2-MSN prolonged stimulation [93]. A vast body of evidence has shown that calretinin- and calbindin-expressing interneurons colocalize in the various corticolimbic regions, yet further research is needed to fully understand the role of calretinin and opioid colocalization in reward and addiction.

### 5.7. Opioids and Neuropeptide Y

The colocalization of neuropeptide Y (NPY) and opioid receptors in the mesocorticolimbic regions shows a functional interaction between these two systems in the regulation of reward and reinforcement. Several studies have investigated the effects of NPY on opioid-mediated reward, and the results suggest a complex interplay between these two systems. For instance, NPY enhances the effects of opioids on dopamine release and reward-related behaviors in animal models. The increase in striatal NPY enhanced the motivational performance followed by a substantial decrease in dopamine; however, NPY injection in the NAc shell decreased the motivational behavior [184]. This enhancement of opioid-mediated reward by NPY receptors is located on dopamine neurons in the mesolimbic dopamine system [185]. Additionally, NPY has been shown to play a role in the development of opioid tolerance and dependence. Chronic exposure to opioids could result in changes in NPY expression and function in the brain, leading to the development of tolerance to the drug’s effect and withdrawal symptoms upon cessation of use [186]. NPY-expressing neurons regulate feeding and reward-seeking behaviors by interacting with serotonergic and dopaminergic pathways of the LHb. NPY neurons innervating to the VTA and the dorsal raphe nucleus enhance emotional eating behavior [187]. We suggest that these reward-seeking behaviors in the LHb and the VTA may be mediated by opioids, whereas distinct studies indicated the importance of these regions in opiate-mediating signaling. The ventricular injection of leptin and NPY affected balancing energy and contributed to the reinstatement of heroin seeking induced by food deprivation [188]. NPY is mainly produced in the arcuate nucleus (ARC) innervating to the paraventricular nucleus (PVN) to modify the mechanisms of food-taking and food-seeking behavior. Activating NPY-expressing neurons in the ARC-PVN pathway via fasting, exercise, and energy loss results in an increase in food intake. Moreover, the expression of NPY in the dorsomedial hypothalamus is enhanced by chronic food restriction in rodent models of obesity. Consistently, an increase in mRNA level of NPY could be observed in food-deprived animals, and NPY-expressing neurons were colocalized with prodynorphin in the hypothalamus since in mice with dynorphin deficiency, NPY expression was strongly downregulated [189,190]. It is postulated that dynorphin induces changes in feeding mechanisms, and the body weight and food intake competence depends on the activity of hypothalamic orexinergic and NPY-expressing neurons [190]. In humans, low expression of NPY is related to negative emotional phenotypes. Functional magnetic resonance imaging of human patients provides evidence that NPY is associated with the salience sensitivity in the bilateral NAc, accounting for the fact that minor differences in NPY expression can modify the state of disorder in the mesoaccumbal function through opioids. In subjects with high levels of NPY, head motion is greater due to the hyperactivity induced by the NPY-expressing neurons [191]. These findings demonstrate the importance of the small changes in the levels of NPY in mood and reward-related mechanisms contributing to the opioidergic system. The results from functional and structural colocalization of opioids and interneurons have necessary implications for developing new therapies for opioid addiction and reward-related disorders. In conclusion, the interaction between NPY and opioids in regulating addiction is a complex and multifaceted process. Further research is needed to uncover molecular and cellular underlying mechanisms.

## 6. Opioids Regulate GABAergic Transmission-Induced Changes in Plasticity

**Figure 1:**
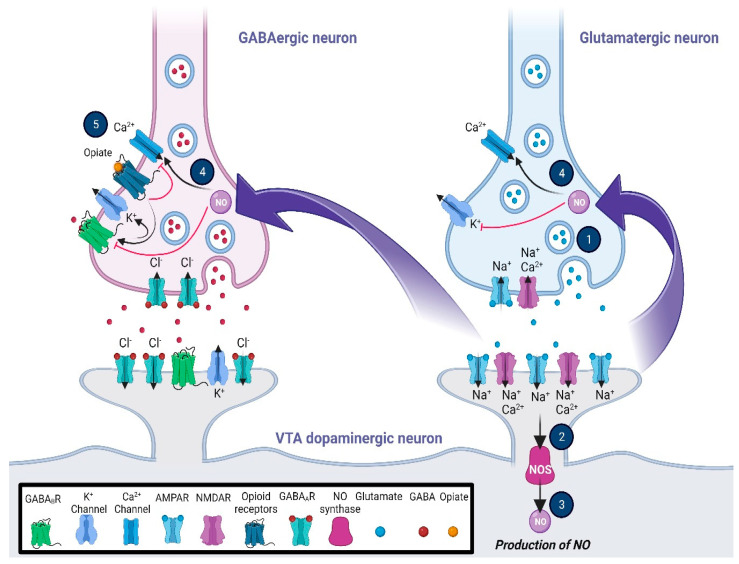
Glutamatergic and opioidergic signaling mediates the induction of synaptic plasticity in GABAergic neurons of the ventral tegmental area (VTA). The LTP of the VTA GABAergic neurons, which is heterosynaptic, may be triggered by the NO–cGMP signaling pathway via the activation of the adjacent glutamatergic neurons. Following the stimulation of NMDA receptors (1), NO as a retrograde messenger (2, 3, and 4) increases the presynaptic GABA release to induce GABA LTP, which may require the balance of neuronal firing in the VTA dopaminergic neurons. On the other hand, activation of the mu-opioid receptors (5), while inhibiting the activation of voltage-gated calcium channels, increases the outflow of K^+^ current via activation of the presynaptic potassium channels. The general effects are blocking GABA release, changing the pattern of action potential, and inducing hyperpolarization in the VTA presynaptic GABAergic neurons. Abbreviations: cyclic guanosine monophosphate, cGMP; gamma-aminobutyric acid, GABA; long-term potentiation, LTP; nitric oxide, NO; N-methyl-D-aspartate, NMDA; ventral tegmental area, VTA.

Similar to the excitatory synapses, LTP/LTD synaptic plasticity can occur in GABAergic neurons in different brain areas through various mechanisms [192]. One interesting study has suggested that LTP of the VTA GABAergic neurons, which is heterosynaptic, may be triggered by the nitric oxide–cyclic guanosine monophosphate (NO-cGMP) signaling pathway via the activation of the adjacent glutamatergic neurons [193]. Following the NMDA receptor stimulation, NO as a retrograde messenger increases presynaptic GABA release to induce GABA LTP, which may require the balance of neuronal firing in the VTA dopaminergic neurons [194]. It is important to note that the activation of the VTA MORs suppresses the GABA LTP (Figure 1). The activation of MORs of GABA neurons attenuates GABAergic synaptic transmission and also GABA LTP, which is for the benefit of glutamate LTP and increases the neuronal firing in the VTA dopamine neurons [195]. Presynaptic potassium channels, β-arrestin2, and proto-oncogene tyrosine-protein kinase Src were suggested to be contributors to mechanisms of MORs mediating the inhibitory GABA release [8,125]. MORs not only regulate the VTA GABAergic transmission at local interneuron synapses but also are involved in the function of the NAc and the ventral pallidum GABAergic inputs [119,196,197]. For example, activation of MORs could inhibit GABAergic inputs from the ventral pallidum onto dopamine and non-dopamine neurons. Opioids differentially inhibit the mesolimbic neurons depending on their target projections [126].

Different amygdala nuclei receive GABAergic inputs from each other and the different brain areas, including the hippocampus, the PFC, and the NAc [198]. The activation of presynaptic MORs in the BLA, the CeA, and the BNST inhibits GABAergic inputs to stimulate these brain areas [199,200]. Similar to MORs, the activation of DORs and KORs inhibits GABA transmission in the BLA and the BNST, respectively [201,202]. These receptors induce the voltage-gated potassium channel (Kv) 1.2 currents to hyperpolarize GABAergic inputs [200]. All three opioid receptors are heterogeneously distributed throughout the entire hippocampus to be activated by endogenous opioids [74,203]. The activation of the CA1 DORs and MORs disinhibits pyramidal neurons to induce LTP [204]. Following the binding of opioids to these receptors, GABAergic neurons were hyperpolarized to reduce their inhibitory action on CA1 pyramidal neurons [133,205]. The location of both receptors is different in GABAergic interneurons. MORs mainly inhibit the interneuron input to the soma, whereas DORs inhibit the input to the dendrites of pyramidal neurons [9]. The stimulation of KORs lacks any effect on the CA1 region. However, the activation of these receptors in the DG produces hyperexcitable granule cells through a postsynaptic G protein-Kv4.2 A-type potassium current mechanism to suppress the LTP induction. The activation of MORs and DORs hyperpolarized the granule cells, which are crucial for the DG LTP induction [206]. Similar to the hippocampus, all opioidergic receptors are expressed in the cortical areas, which mediate memory formation, rewarding, and emotional processes [207,208,209]. Interestingly, DOR activation disinhibited and hyperpolarized the ACC pyramidal neurons, while MOR activation hyperpolarized the non-pyramidal cells and suppressed excitatory thalamic inputs in the ACC [210,211]. In the mPFC, the activation of DORs increases GABA transmission from somatostatin-expressing interneurons to PV-expressing interneurons, which disinhibits pyramidal neurons (Jiang et al., 2021). The activation of MORs, DORs, and KORs inhibits glutamatergic transmission to the mPFC to induce glutamatergic LTD [212,213,214].

## 7. Intracellular Mechanism Underlying Opioid–GABA Crosstalk

In the CeA, the activation of KOR signaling by dynorphin results in a reduction in presynaptic GABA release following alcohol intake [215]. Notably, neurons projecting from the periaqueductal gray to the CeA exhibit MOR-sensitive GABAergic input [194]. In addition, KORs exert a tonic inhibition on GABAergic inputs to the CeA identical to MORs and DORs [216]. The opioidergic system not only regulates GABAergic transmission, but also regulates glutamatergic transmission indirectly through GABAergic modulation. Glutamatergic inputs to the CeA corticotropin-releasing factor (CRF) neurons are not sensitive to KORs, although the activation of KORs presynaptically blocks local GABAergic neurons receiving inputs from the parabrachial glutamatergic neurons, following disinhibition of the CRF-projecting neurons [217]. Activation of KORs increases the presynaptic GABA release in the BLA caused by the dose-dependent exposure to tetrodotoxin (TTX) [216]; in contrast, the enhancement in the activity of receptors decreases the release probability in neurons projecting from the lateral amygdala to the BLA, following an inhibition in the induction of LTP. The activation of KORs has different impacts on GABAergic cells, resulting in depression, potentiation, or no response [202]. Moreover, MOR-expressing neurons in the ventrolateral BNST presynaptically block GABA release onto the VTA-projecting neurons [218]. These findings have addressed numerous examples of the crosstalk between GABAergic and opioid receptors; however, there is not enough evidence accounting for the crosstalk between opioids and the GABAergic system in the reward-related mechanism of the corticolimbic subregions, specifically in the amygdala.

### 7.1. Intracellular G Protein Involvement in Opioid/GABA Reinforcement

**Figure 2:**
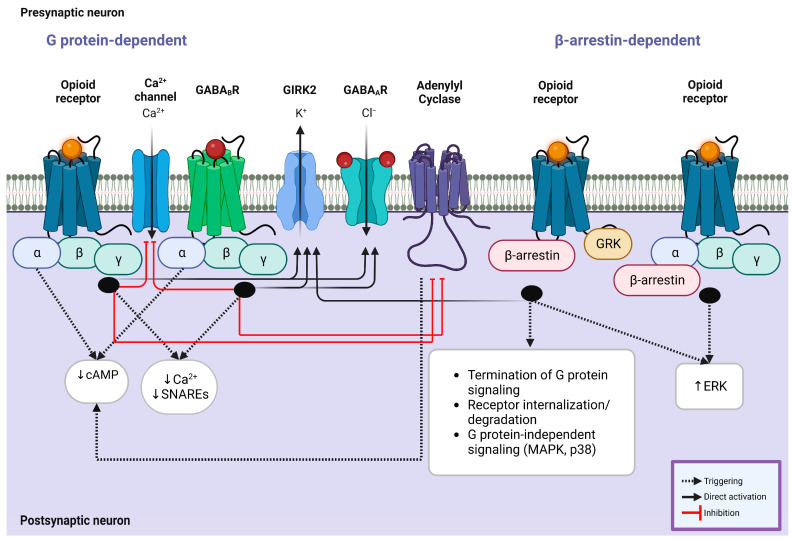
Intracellular G protein involvement in opioid/GABA crosstalk. (**Left**) Coupling of opioid receptors with their endogenous or exogenous ligands and GABA_B_ receptors with GABA results in the simultaneous inhibition of calcium ion channel influx and enhancement of GIRK2 and GABA_A_ receptors, leading to a significant reduction in the production of cAMP and decreased calcium response mediated by Gα_i_ subunit. This mechanism is modulated by known kinases and phosphorylases, including adenylyl cyclase, GRK, ERK, and PKC, which contribute to the synergistic effect of opioid agonists on the suppressive effect of GABA receptors. (**Right**) Opioid receptors also exert their impact via β-arrestin-mediated mechanisms. GRK phosphorylates the receptor following the recruitment of β-arrestin, which causes receptor desensitization and internalization. Similarly, this mechanism improves the activity of GIRK2 channels and regulates the signaling of MAPKs (p38) and ERK1/2 through the Gα_i_ subunit, which is shared with the downstream cascade of GABAergic receptor signaling. Abbreviations: cAMP, cyclic adenosine monophosphate; SNARE, soluble N-ethylmaleimide-sensitive factor attachment protein receptor; GABA, gamma amino butyric acid; GIRK, G protein-gated inwardly rectifying potassium channel; GRK, G protein-coupled receptor kinase; MAPK, mitogen-activated protein kinase; ERK, extracellular signal-regulated kinase.

In the downstream cascade of MORs, KORs, DORs, and NOPRs, a decline in cAMP and protein kinase A levels via the decrease in adenylyl cyclase activity results in the opening of potassium channels and closing of the calcium channels (Figure 2). This mechanism can modulate synaptic plasticity in different levels and memory formation during motivational and reward-related tasks [15,219]. On the other hand, GABAergic receptors also potentiate the intracellular signaling pathways to inhibit neuronal activity by opening the chloride channels following hyperpolarization along with binding to the TrkB receptors of neurons triggering a more substantial silencing effect [220]. This mechanism is mediated by intracellular G proteins akin to opioid receptors; it involves activating the downstream effectors such as adenylyl cyclase, phospholipase C, and protein kinase C (PKC) [221], and therefore, it is expected that shared mechanisms of both opioidergic and GABAergic activation of downstream GPCRs lead to the changes in reward outcomes (Figure 2; Table 2). The widespread activity of GPCR types of opioid receptors is evident in the corticolimbic regions. Activation of KORs and DORs in the CA3 region as a principal hub in the hippocampal network does not change the IPSPs; however, MORs regulate GABA release by blocking PKC activation, which consequently provokes a G protein-mediated disinhibition. Intriguingly, these changes do not involve any alteration in the conductance of calcium or potassium [106,135]. In the CA1 region, MORs and DORs separately enhance the activity of G protein-coupled inwardly rectifying potassium channels in PV-expressing neurons, which results in membrane hyperpolarization and limits GABA release onto the pyramidal cells (Figure 2) [114]. While the activation of GPCRs through opioids and GABA is evident, this alteration may lead to reward and learning reinforcement. It is widely accepted that the NAc core and the shell compartments contribute to the reinforcement via interaction with reward circuits in the conditioned tasks [222]. In the CPP task, cocaine administration in female mice shows different levels of salience and reinforcement along with changes in GABAergic transmission in preference for the environments containing lower morphine dosage compared to males, which reveals the sex differences in opiate reinforcement and reward [223]. Reinforcing behavior can be a result of food intake and fat absorption. The importance of reinforcing behavior in addiction and addiction-related disorders such as chronic obesity is clear to all individuals investigating these mechanisms. Numerous pieces of evidence have demonstrated that the mesolimbic striatal complex and dopaminergic system are associated with feeding and food-related reward behaviors, which the NAc also mediates as one of the key regulators of the reward system. This modulation is regulated by GABAergic neurons with or without the expression of opioid GPCRs, which results in changes in dopaminergic inputs to the NAc [224]. Another study focusing on the reinforcement in food intake manifested that ghrelin/GHS-R1A signaling may be involved in the reinforcing effect of opioid GPCRs in addiction [225]. The excitability of MORs in the NAc is associated with dopamine efflux owing to the suppression of the release of GABA from the VTA interneurons following a tonic inhibition in the mesocorticolimbic dopaminergic neurons [226]. These results shed light on the importance of GABAergic and opioidergic interference in reinforcement and reward circuitry.

### 7.2. Opioidergic/GABAergic Intracellular Signaling Pathways

Endogenous and exogenous opioids can diversly affect the intracellular signaling of GABAergic neurons, mainly through GPCRs; however, various studies have proven that opioids occasionally mediate their effects through non-GPCR channels or receptors. For instance, β-arrestin2 pathways and K^+^ ion channels have been commonly indicated in opioids mediating reward (Figure 2) [17]. In the cortical regions, MOR-expressing neurons inhibit GABAergic interneurons across membrane hyperpolarization by increasing the conductance of potassium [175]. The inhibitory impact of KORs presynaptically regulates GABAergic neurons of the CeA projecting to the BNST. This regulation is controlled by extracellular signal-related kinases (ERKs) (Figure 2). Noteworthily, p38 signaling compartments do not contribute to the aforementioned suppressive activity of KORs [137]. Conversely, the enhancement of KOR activity in the BNST GABAergic neurons innervating to the BLA triggers presynaptic LTD through p38 signaling downstream proteins independent from MAPK- and protein kinase A-related pathways following modulation in calcium signaling in the BNST (Figure 2), whereas this modulation is differently relayed in the PFC inputs. The net impact of KOR modulation is a significant decline in the action potential firing of the BNST neurons despite the KOR-induced inhibition of GABA transmission [201]. It is a compelling fact that glutamatergic metabotropic receptors can also undergo the modulation of opioid-mediated GABAergic transmission in the lateral geniculate nucleus of the thalamus. Parvocellular neurons of this region experience LTD induced by GABAergic inputs following L-type calcium channel-dependent activity of mGluR5 in enkephalin released from somatodendritic synapses to exert effects on presynaptic MORs. Long-term activation of MORs via this mechanism is required to induce LTD, which can be reversed by administering naloxone [235]. Occasionally, opioidergic receptors modify receptor internalization and expression. The VTA DOR-expressing neurons acquire the ability to generate a postsynaptic infusion of GABA_A_ receptors in a subpopulation of neurons through Akt and phosphoinositide 3-kinase signaling [142]. It is relevant to state that DORs do not associate with the synaptic inputs of GABAergic neurons of the rostromedial tegmental nucleus innervated from the lateral habenula [196]. In the cortex, acetylcholine receptors can be regulated by opioid receptors mediating GABAergic inputs. Nicotinic acetylcholine receptors undergoing opiate regulation trigger GABAergic input to pyramidal cells following a primary increase in IPSCs resulting in a decrease to a level lower than the baseline levels. Remarkably this reduction is regulated by MOR antagonists, suggesting that the activation of nicotinic acetylcholine receptors causes enkephalin release for feedback control [236]. In the dorsal striatum, one of the targeted areas of the basal ganglia for reward-related learning, KORs mediate the inhibition of GABA transmission via calcium influx following changes in the activity of N-type voltage-gated calcium channels (VGCCs) and not the inhibition of potassium channels [237]. Upon the activation of dopamine D2 receptors and GABA receptors, GIRK is opened following the regulation of adenylyl cyclase [238]. Colocalization of GABA_B_, Kir3, and D2 receptors can strengthen the hypothesis that these receptors contribute to processing reward-related mechanisms (Figure 2). It is well established that the function of Kir3 channels is associated with the non-analgesic effects of opioids. For instance, the dampening of the activity of the DG GABAergic neurons by the activity of MOR and DOR agonists involved in voltage-dependent K^+^ channels and the Kir3 mechanism enhances the excitability in glutamatergic granule cells, which results in a reduction in seizure threshold and increased severity [95,239,240]. Divergent regulation of GABA_B_ receptors leads to Kir3 and GABA_B_ receptor internalization in the VTA following changes in reward output. It is postulated that this crosstalk and these shared mechanisms are evident in non-pathological conditions of the hippocampus related to the reward system since opioids lower the excitability of DG granule cells in reward mechanisms [136].

## 8. Inhibitory Mechanisms of Reward–Aversion in Addiction

The altered activity of GABA receptors and opioid abuse may be associated with mood disorders. McHugh and coworkers (2021) have shown that anxiety responding to distress is elevated in patients suffering from opioid use disorder which is associated with the severity of misuse and appears to amplify risk via an increase in relapse and seeking for drugs [241]. Comorbidity of opioid use disorder, anxiety, and depression could impact the treatment outcomes [242]. Therefore, understanding the mechanisms involved in the association of opioid use disorder, GABA dysfunction, and mood disorder is highly recommended, and aversion therapy may have beneficial impacts. It seems that a functional reduction in dopaminergic neurons and an increase in the cholinergic neurons’ activity in the brain reward system trigger aversion. It should be considered that opioids have modulatory effects on reward and aversion processes [243]. The use of pharmacotherapy, including consumption of methadone, buprenorphine, or injectable naltrexone, in combination with psychosocial support can alleviate the symptoms, but there are varying retention rates for different pharmacological drugs. Cotreatment with buprenorphine, as an opioid receptor partial agonist, and naloxone, as an opioid receptor antagonist, could induce the aversive effects in opioid use disorder [244]. Naltrexone, an opiate receptor antagonist, blocked the pleasant and reinforcing effects of alcohol by preventing the stimulation of opioid receptors and decreasing the dopamine release in the VTA. It seems that naltrexone has an anti-craving impact on alcohol use disorder [245].

Prolonged exposure to opiates modifies the neuronal circuits to contribute to adverse effects and the antireward state. Changes in inputs to the NAc are one modification involved in the aversion associated with opiate withdrawal [246]. For example, chronic morphine increased the excitatory input to the NAc D2-MSNs via inserting GluA1-containing AMPA receptors. Inhibition of this activity reversed the somatic symptoms of morphine withdrawal, and naloxone precipitated the withdrawal place aversion [247]. Additionally, the activation of KORs inhibited the VTA dopaminergic neurons to induce aversive responses [248]. Hence, KOR antagonists represent a novel therapeutic approach to restoring circuit function under drug abuse. In addition, receptor downregulation, signaling desensitization, and upregulation of drug metabolism trigger opioid tolerance as a reduction in drug potency to induce aversion [249]. GABAergic drugs such as topiramate and baclofen also target the NAc inputs and cause aversion. These drugs reduce the DA release in the NAc, and thereby motivational and reinforcement effects of drug abuse can be used as a treatment for drug dependency [250]. For example, the activation of GABA_B_ receptors reduced GABA signaling during opioid withdrawal and reduced the withdrawal symptoms [251]. In general, this may suggest that the combination of opioid receptor antagonists and GABAergic drugs may enhance the aversion and lessen the withdrawal effects in opioid use disorder patients.

## 9. GABAergic/Opioidergic Systems Modulating Feedback and Feedforward Activity

GABAergic inhibition in local corticolimbic circuits potentiates feedback and feedforward inhibition on adjacent excitatory or inhibitory neurons. Accordingly, many of them express opioid receptors to mediate the net inhibition induced by GABAergic neurons. In the hippocampus and the cortex, interneurons expressing MORs control the excitability of the feedforward inhibition. Activating hippocampal MORs may lead to suppressing feedforward inhibition, which results in driving gamma rhythm and, consecutively, diminishing phase coupling in gamma oscillations between the CA1 and the CA3 regions [9,61,73,99,164]. In the connection between the entorhinal cortex and the hippocampus, during field gamma oscillation, the active pyramidal cells in the superficial layer of the entorhinal cortex (L3) generate tonic excitatory inputs to the subset of interneurons characterized as stuttering cells, which results in feedback inhibition; however, the activation of MOR-expressing parvalbumin interneurons leads to the disinhibition or feedback excitation of stuttering cells and elevation of gamma frequency oscillation via pyramidal neurons which generate gamma oscillations in the active network [252]. It is worth knowing that the feedforward mechanism is propagated from the pyramidal neurons, mainly projecting from superficial cells, while feedback modulation forms from the deeper pyramidal neurons innervating to the superficial layers of cortical areas such as the auditory cortex [253]. Moreover, the activation of acetylcholine receptors induces gamma frequency oscillation in vitro following the potentiation of the pyramidal interneurons’ gamma mechanism. Pyramidal neuron excitation in the pyramidal interneuron gamma model triggers the firing of local interneurons, which results in the feedback inhibition of pyramidal neurons, and this cycle reoccurs after the inhibition decays [252]. In addition, MOR activation endogenously reduces the stress exposure facilitated by LTD in the elevated plus maze (EPM) by lowering the feedback and feedforward inhibition rate on the pyramidal neurons of the CA1 stratum oriens and stratum lacunosum moleculare [133,148]. The amygdala intercalated cells (ITCs) are responsible for emotional information flow in this region. These densely packed GABAergic neurons generate feedforward inhibition in the CeA and the BLA inputs/outputs projecting from the thalamic and cortical areas [254]. The ITC-induced feedforward inhibition can also modulate the inputs to the dorsal striatum as a region contributing to the reward-prediction-error system [255]. Accordingly, the activation of MORs in the ITCs takes part in reward salience. In rats, opiate withdrawal reduces the feedforward inhibition by the ITCs in the BLA and strengthens the inputs of the BLA, projecting neurons to the NAc and changing the reward system [256]. GABAergic inhibitory neurons expressing MORs in the lateral paracapsular part of the BLA exert feedforward inhibition on the excitatory pyramidal neurons, contributing to anxiety-like behavior and stress-induced drug seeking [143]. It is remarkable that medial ITCs expressing MORs similarly regulate the gating of information flow by triggering feedforward inhibition on the medial part of the CeA pyramidal neurons of the BLA to the CeA pathway [257]. Notably, thalamic–corticostriatal inputs are not exempt from the opioidergic regulation of GABAergic neurons inducing feedback or feedforward inhibition. MSNs innervating the pyramidal neurons of the ACC from the medial thalamus broadly express opioid receptors. The activation of MORs on these neurons inhibits the inputs to ACC. However, the activation of DOR-expressing neurons disinhibits the ACC pyramidal neurons by suppressing local feedforward inhibition by PV-expressing interneurons. This result accounts for the polysynaptic facilitation of excitation by DOR-expressing neurons in thalamic–corticostriatal inputs [210]. It has been postulated that GABAergic neurons contributing to the emotional response in the ventrolateral periaqueductal gray block the activity of neurons in the dorsal raphe nucleus through feedforward inhibition. This pathway may modulate anti-anxiogenic behavior in the presence of morphine as an opiate in mice [258].

## 10. Opioids and GABA in Network Oscillations

Rhythmic changes in cell membrane potentials following repetitive hyperactivity generate field potential oscillations and oscillatory activity. Oscillation orchestrates the activity locally or in distant regions of the brain. Oscillatory activity in hippocampal ensembles occurs between two common theta and gamma oscillation ranges. The modulation of muscarinic acetylcholine receptors (mAchRs) has an essential and causal role in regulating oscillation in the theta band and rhythm generation [259]. It is thought that rhythmic inhibition through PV-expressing interneurons is crucial for producing oscillation in the hippocampal CA1 region in Ach-mediated oscillations. Interestingly, PV-expressing interneurons involved in this modulation express MORs to moderate the oscillatory activity evoked by GABAergic and cholinergic neurons, suggesting that the mAch-induced inhibitory rhythmic IPSCs in the CA1 region are modulated by FSI-expressing MORs [73,260,261]. In the PFC region undergoing heroin addiction, the impairment of information flow recorded by EEG during tolerance and withdrawal causes the desynchronization of oscillatory activity in the lower alpha rhythm via glutamine–GABA imbalance [262]. In addition, reward-related pathways of the brain intervene in the functional output of the NAc and control the excitation and inhibition balance. The FSIs of this pathway exert the most robust inhibition of the NAc neuronal outputs leading to changes in network oscillation. Each FSI in the NAc projects its axon to the hundreds of principal neurons to control the activity via feedforward inhibition [263]. The oscillatory activity in neuronal ensembles involved in the reward system can be generated and regulated by the different activity of ion channels. L-type VGCCs are expected to be active during the oscillatory activity of neurons. The synchronous activity of calcium transients is regulated by this type of channel in physiological and epileptiform conditions. In the striatum, the activity of GABAergic and opioidergic GPCRs may indirectly influence the activity of the VGCCs. The sensitivity of Ca^2+^ and probability of dopamine and GABA neurotransmitter release depends on proximity to VGCCs; thus, we postulate that the oscillatory activity under the interactive activity of GABAergic and opioidergic systems in the reward mechanism may be identically mediated by the ensuing malfunction of the VGCCs and their functional interaction with GABAB receptors. Yet, extensive research is required to confirm this mechanism [264,265]. Not only neurons but also astrocytes may participate in network oscillation induced by interneurons under opioidergic modulation. Astrocytes express opioid receptors in response to opioids released from GABAergic interneurons [266]. Initial activation of SST-expressing interneurons can evoke a strong and long-lasting calcium elevation following oscillations under repetitive activation in astrocytes. The astrocytic network can maintain the sustained activity of SST-expressing interneurons. In this mechanism, the calcium incline in astrocytes, mediated by GABA_B_ and opioid receptors, is altered in response to the repetitive activation of PV- or SST-expressing interneurons, suggesting that the plasticity of astrocytes depends upon the interneuron subtypes and their activity [267]. A subset of neuronal ensembles in the amygdala repeatedly fires, leading to network oscillations. This rhythmic activity triggered by ensembles plays a significant role in fear memory. As previously described, ITCs expressing opioid receptors broadly contribute to this network’s oscillatory patterns by firing discretely from hippocampal CA1 theta and gamma oscillations or cortical activity, accounting for ITCs not orchestrating the network oscillation between various brain regions. Interestingly, spike durations recorded from GABAergic neurons and ITCs are comparable, meaning these cells closely coordinate the network activity [268].

## 11. Modulation of GABAergic/Opioidergic Transmission in Pathology and Therapeutics

During developmental processes, GABAergic transmission has a modulatory role in neural proliferation, migration, differentiation, growth, and synaptogenesis [269,270]. In addition, adult brain inhibitory neurons regulate neural network activity by controlling circuit connectivity and dynamics [271]. GABAergic signaling dysfunction leads to an imbalanced excitatory/inhibitory transmission in neuronal activity, thereby contributing to several neurodevelopmental disorders, including schizophrenia [272,273], autism spectrum disorders [274], fragile X [275], and Tourette syndrome [276]. Genetic studies have revealed an association of blood expression of GABA_A_ receptor subunits and GABA_A_ receptor-associated protein with the symptom severity of Tourette syndrome [277]. Increased GABAergic transmission [278] and GABAergic markers such as GAD65 and GAD67 [279] were reported in a mouse model of Down syndrome. Therapeutic strategies are mainly aimed at targeting GABA_A_ receptors or GABA metabolism. Several preclinical studies have confirmed the possible benefit of GABA_A_ receptor agonists for ameliorating autistic-like behavior [280] and fragile X syndrome [281] as well as a GABA_A_ receptor antagonist as a potential therapeutic agent for cognitive impairment observed in a mouse model of Down syndrome [282]. Given the side effects induced by the administration of GABA_A_ receptor antagonists, developing drugs selectively targeting specific subunits of GABA_A_ receptors will be a more useful pharmacological therapy without adverse effects [283]. For example, in an animal model of Down syndrome, it was found that applying a selective antagonist at the GABA_A_ receptor α5 subunit reversed spatial memory deficits without pro-epileptic side effects in mice [279]. Interestingly, environmental enrichment (EE) through altering GABAergic neurotransmission can be considered a non-invasive approach in neurodevelopmental disorder therapy [284]. EE results in dynamic changes in PV-expressing interneurons of the hippocampal CA2 region, which causes plasticity to affect cognitive functions. EE reduced the DOR-mediated plasticity in the CA2 area [285]. Opioidergic transmission has a neuromodulatory role in striatal projection neuron activity and motor control, and MOR signaling malfunction results in pathological conditions, including Parkinson’s disease [286]. Observation showed that the drugs targeting MORs could be useful pharmacological interventions to treat dystonia [287]. Dysfunction of KOR signaling leads to the emergence of positive, negative, and cognitive symptoms of schizophrenia [288]. These data suggest that manipulating these receptors can be considered a new therapeutic option. Since DORs are involved in controlling the dorsal hippocampal–striatal balance, DOR knockout mice presented impairment of dorsal hippocampus-dependent tasks, including associative learning, when the balance was biased towards facilitated striatal function [289]. Alteration of opioid receptor signaling increased gene expression implicated in Aβ production, thereby contributing to Alzheimer’s disease (AD) pathogenesis [290]. In conclusion, the results mentioned above make GABAergic and opioidergic signaling pathways a promising clinical perspective for the therapeutic purpose of neurodevelopment/neurodegenerative diseases.

## 12. Discussion

The functional interaction between GABAergic and opioidergic systems in the corticolimbic regions indicates their pivotal effects on memory formation, reward, anxiety, depression, and pain processing. In this review, we highlighted the importance of understanding how these systems mediate reward/reinforcement and how the incidence of dysfunction in GABAergic and opioidergic interaction affects the induction of synaptic plasticity. Previous findings have suggested that opioidergic transmission, mainly through presynaptic MORs, increases GABA release in the VTA and decreases it in the basal ganglia [28], the NAc [54], the hippocampus, the amygdala, and the PFC [6,7,46] while increasing dopamine release in the striatum and decreasing it in the VTA and the NAc [27]. This dampening effect on dopaminergic signaling is thought to contribute to the analgesic and anxiolytic effects of opioids, although chronic abuse of opioids leads to neuroadaptation, which is believed to contribute to the development of opioid tolerance, dependence, and withdrawal following an increase in dopamine release in the NAc and enhanced GABAergic transmission. Accordingly, we assume that opioidergic modulation has a suppressive impact on the majority of the corticolimbic brain regions associated with reward-related behaviors, while it can modulate dopamine levels differently. This review explains the interactive role of these systems exclusively in the reward-related brain regions, whereas previous findings have solely identified the role of GABAergic or opioidergic systems in reward modulation. It is important to note that the shared molecular mechanisms of GABAergic and opioidergic receptors are essential for the rewarding properties of the stimuli and cognitive aspects of motivational behaviors in the corticolimbic regions. We also discussed the colocalization of opioid and GABA receptors in the corticolimbic regions to extend the current knowledge of the coexpression and colocalization of these two systems. Opioid receptors vastly colocalize with various subtypes of GABA receptors in different corticolimbic areas, suggesting that their interactive effect is important to investigate in clinical applications. Given that the shared mechanisms between opioidergic and GABAergic systems are pivotal, therapeutic approaches should consider the interactive roles of these systems in the treatment of depression, anxiety, and mood-related disorders. The concept of opioid–GABA interactions represents a novel approach to the development of new therapeutics for addiction and reward-related disorders. By targeting the interactions between these two systems, it may be possible to develop more efficacious and targeted treatments.

## 13. Limitations

Although the corticolimbic signaling pathways of GABAergic and opioidergic transmission have been widely studied and understood, there are several limitations that need to be considered. To begin with, these systems have been independently studied, and only a few studies have indicated the shared mechanisms. Furthermore, while our understanding of the corticolimbic reward system has improved, it can be challenging to draw definitive conclusions about the region-dependent functioning of these systems and their interconnection between various subregions of corticolimbic areas. In addition, research on opioidergic and GABAergic interaction in the amygdala and the PFC is lacking and requires further studies to address the role of these systems in modulating motivational behaviors. Although opioids can modulate GABAergic transmission by presynaptic inhibition or postsynaptic modulation, they can also exert different effects on GABAergic subtypes and circuits depending on their concentration or duration of action. Additionally, studies on the importance of endogenous opioids in modulating reward systems in GABAergic neurons are scarce compared to studies on exogenous opiates. It should be considered that research on several types of opioid receptors, especially KORs and DORs, is insufficient, while most studies have focused on MORs regulating GABAergic transmission in corticolimbic regions. Finally, it should be considered that many of the studies on GABAergic and opioidergic neurons have been conducted in animal models, and it is not always clear how well these findings translate to humans; therefore, continued research in this area is essential for advancing our understanding of the functioning of the brain and developing new treatments for a range of neurological and psychiatric disorders.

## 14. Concluding Remarks

Investigating the importance of opioidergic modulation in GABAergic synapses is worthwhile in understanding reward-related and motivational behaviors. This modulation plays a crucial role in motivation and addictive behaviors in the corticolimbic regions, as shown in Table 2. It can be inferred that opioidergic and GABAergic synapses work together to regulate the activity of the NAc and the VTA dopaminergic neurons, thereby triggering synaptic plasticity and subsequently modulating reward mechanisms through shared signaling pathways. The interaction between receptors may result in the production of brain-derived neurotrophic factor and the regulation of plasticity-related genes, including immediate early genes necessary for LTP or LTD induction. At the behavioral scale, the interactive role of GABAergic and opioidergic transmission alters dopamine release in the reward system, and opioids modulate the excitability of this system by controlling GABA release; therefore, this modulation changes the behavioral outcome. At the cellular level, GABAergic neurons expressing opioid receptors interact with adjacent cells, while the receptors of these neurotransmitters share common mechanisms by modulating ion channels and metabotropic GPCRs (Table 2). This ultimately regulates neuronal excitability through retrograde signaling and neurotransmitter release. At the molecular level, they interact through downstream signaling pathways of specific kinases and phosphatases (Figure 2). Understanding the shared mechanisms between GABAergic and opioidergic neurons has significant implications for developing novel therapeutic strategies for motivational and depressive-like behaviors.

## Figures and Tables

**Table 2:** Shared common intracellular signaling pathways of opioidergic and GABA_B_ receptors.

Protein/Cascade	GABA_B_ Receptors	Opioidergic Receptors
The activity of adenylyl cyclase	Inhibition [227]	Inhibition [228]
Cyclic adenosine monophosphate levels	Reduction [227]	Reduction [228]
The activity of GIRK channels	Enhance [227]	Enhance [228]
The activity of voltage-gated Ca^2+^ channel	Inhibition [227]	Inhibition [228]
The activity of voltage-gated Na^+^ channel	Inhibition [229]	Inhibition [62]
The activity of phospholipase C	Enhance [230]	Enhance [55]
Secretion of brain-derived neurotrophic factor	Increase [230]	Increase [55]
P38/MAPK signaling	Enhance [230]	Enhance [55]
The activity of ERK	Enhance [231]	Enhance [232]
The activity of phosphorylated JNK	Enhance [233]	Enhance [234]

Abbreviations: G protein-coupled inwardly-rectifying potassium channel, GIRK; mitogen-activated protein kinase, MAPK; extracellular signal-regulated kinase, ERK; c-Jun N-terminal kinase, JNK.

## Data Availability

Not applicable.
